# Optimal fertigation for high yield and fruit quality of greenhouse strawberry

**DOI:** 10.1371/journal.pone.0224588

**Published:** 2020-04-01

**Authors:** Yong Wu, Li Li, Minzan Li, Man Zhang, Hong Sun, Nikolaos Sigrimis

**Affiliations:** 1 Key Laboratory of Modern Precision Agriculture System Integration Research, Ministry of Education, China Agricultural University, Beijing, China; 2 Key Laboratory of Agricultural Information Acquisition Technology, Ministry of Agriculture and Rural Areas, China Agricultural University, Beijing, China; 3 Department of Agricultural Engineering, Agricultural University of Athens, Athens, Greece; Institute of Soil Science, CHINA

## Abstract

Nitrogen (N), phosphorus (P), potassium (K), and water are four crucial factors that have significant effects on strawberry yield and fruit quality. We used a 11 that involved 36 treatments with five levels of each of the four variables (N, P, and K fertilizers and water) to optimize fertilization and water combination for high yield and quality. Moreover, we used the SSC/TA ratio (the ratio of soluble solid content to titratable acid) as index of quality. Results showed that N fertilizer was the most important factor, followed by water and P fertilizer, and the N fertilizer had significant effect on yield and SSC/TA ratio. By contrast, the K fertilizer had significant effect only on yield. N×K fertilizer interacted significantly on yield, whereas the other interactions among the four factors had no significant effects on yield or SSC/TA ratio. The effects of the four factors on yield and SSC/TA ratio were ranked as N fertilizer > water > K fertilizer > P fertilizer and N fertilizer > P fertilizer > water > K fertilizer, respectively. The yield and SSC/TA ratio increased when NPK fertilizer and water increased, but then decreased when excessive NPK fertilizer and water were applied. The optimal fertilizer and water combination were 22.28–24.61 g plant^-1^ Ca (NO_3_)_2_·4H_2_O, 1.75–2.03 g plant^-1^ NaH_2_PO_4_, 12.41–13.91 g plant^-1^ K_2_SO_4_, and 12.00–13.05 L water plant^-1^ for yields of more than 110 g plant^-1^ and optimal SSC/TA ratio of 8.5–14.

## Introduction

Mineral fertilizers and water have a significant effect on crop yield [1–3]. However, excessive application of fertilizers may lead to soil and water pollution and become a serious threat to food safety [4,5]. Meanwhile, water scarcity is now a major challenge in China [6]. Therefore, a good management of fertilization and water is increasingly required for agriculture in China.

Strawberry is one of the most profitable fruit cultivars in China, which ranks first in total strawberry production worldwide with a production of 1,801,865 tons in 2016, followed by United States and Mexico among 79 countries [7]. Thus, large amounts of fertilizers and water are necessary for strawberry production in China. Consumers prefer strawberries with sweet taste [8,9], which is effected by the balance between the soluble solid content (SSC) and titratable acid (TA) in ripe fruits [10,11], which are standard quality indexes to assess sweetness and sourness [12]. The ratio SSC/TA is an effective parameter to determine fruit flavor [13,14]. The higher the SSC/TA ratio, the sweeter the fruit [15]. Therefore, increasing strawberry production and enhancing fruit quality with high SSC/TA ratio and without environment pollution are important goals in strawberry growing.

Nitrogen (N), phosphorus (P), and potassium (K) are primary mineral fertilizers. N is the most limiting nutrient to crop production because of its important role in cell division [16,17], and N deficiency can decrease crop yield and quality [18,19]. P nutrient is essential for photosynthesis [20], and it is required after emergence [21]. K is the second most abundant element in plant tissues after N [22,23], and it helps enhance water uptake and fruit quality [24]. In addition to mineral fertilizers, water greatly contributes to the development of the strawberry fruits, leaves and othe organsf [25], and water shortages can lead to large losses of strawberry yield [26].

Although studies have shown that all mineral fertilizers (N, P, and K) and water have effects on the yield and quality of strawberries, most of them only focused on either the effect of water [26–28] or the effect of fertilization [29–33]. The combined application of N, P, and K (NPK) fertilizers and water for high yield and good fruit quality is rarely reported [27,31,34]. Thus, a closer examination of the interaction effect among N, P, and K fertilizers and water on the strawberry yield and fruit quality would be of interest. This study aims to evaluate this interaction and suggest an optimal fertilization and water combination for high yield and good quality.

## Materials and methods

### Experimental site and cultivar

**Fig 1:**
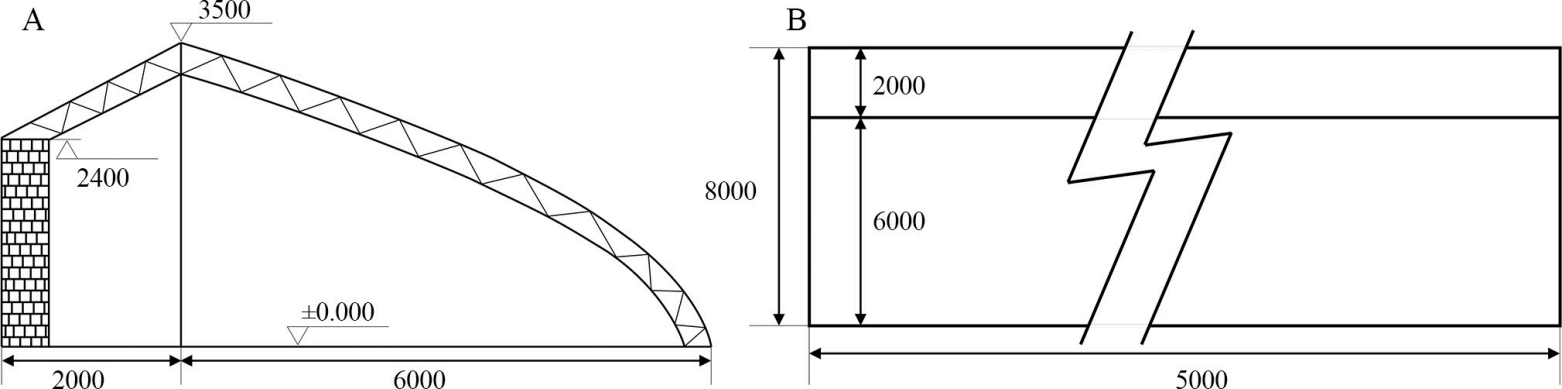
Solar greenhouse block diagram. (A) Section view. (B) Top view.

The experiments went on for 8 months from November 2016 to June 2017 in an east–west oriented solar greenhouse located in the Zhuozhou Experiment Center, China Agricultural University, China. The Chinese solar greenhouse, as a horticultural facility, is a kind of mono-slope greenhouse that provides effective energy use and is widely used in China, especially in the northern latitudes [35,36]. The structure of this solar greenhouse has a typical width, length, backwall height, and roof height of 8, 50, 2.4, and 3.5 m, respectively (Fig 1).

**Fig 2:**
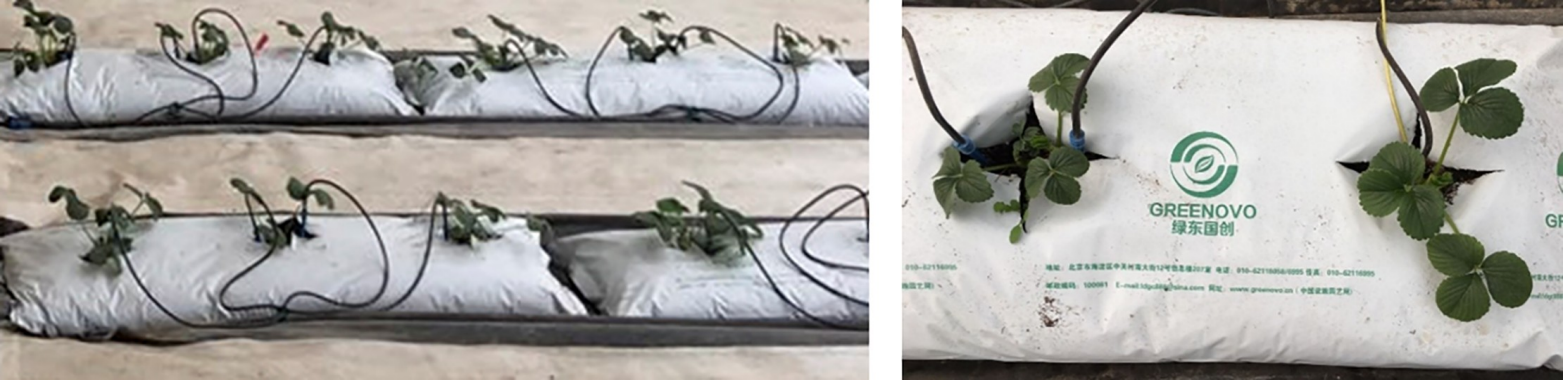
Strawberry plants grown on substrate bags.

The strawberry cultivar used was Hongyan, which has been studied extensively in China [37–40]. It was cultivated in substrate instead of soil in the solar greenhouse with natural light and temperature of 10°C–26°C. The substrate (Table 1) was a mixture of peat, vermiculite, and perlite with a mixture ratio of 10:2:1. The substrate bag (Beijing Greenovo Agriculture Science and Technology Co., Ltd.) was 100 cm×40 cm×20 cm, and three strawberry plants grew in each bag (Fig 2). The strawberries were transplanted on November 3, 2016 and hand-harvested at the mature red stage, which is from late February 2017 to late May 2017, thereafter transported to the laboratory within 2 hrs using ice bags for cooling.

**Table 1:** Substrate chemical analysis (%).

Properties	Fertility
Total Nitrogen	0.97
Total Phosphorus	0.47
Potassium	1.07

### Experimental statistical method

Since this research involved 4 factors with each at five levels, which will results in a total of 1024 treatments according to a full factorial design and orthogonal array[41], a orthogonal rotation central combination design was applied, this regression technique is currently the most effective method for multi-factor interaction effect analysis, which will considerably reduce experiment times without losing efficiency[42][43]. To reveal the relationship between the NPK+water combination and the fruit yield and achieve the optimal combination, a four-factor quadratic regression orthogonal design table was chosen, and five levels (-γ, -1, 0, 1 and γ) were determined for each factor (Table 2)[41] according to the regression orthogonal rotation central combination design, γ is the maximum level for each factor. Assume that *m* (*m =* 4 In this case) indicates the number of factors, *x*_*j*_ *(j = 1*,*2*,*…*, *m)* indicate the independent variables (the four factors), and y indicates the dependent variables (yield and SSC/TA ratio), then the quadratic equation can be defined as follows[44]:

$$y=a+\sum_{j=1}^{m}b_{j}x_{j}+\sum_{k<j}b_{kj}x_{k}x_{j}+\sum_{j=1}^{m}b_{jj}x_{j}^{2},k=1,2,\ldots ,m-1\;(j\neq k)$$ (1)

Where *a*, *b*_*j*_, *b*_*kj*_, *b*_*jj*_ are the regression coefficients to be determined, the total number of which is $1+m+\frac{m(m-1)}{2}+m=\frac{\left(m+1)(m+2\right)}{2}$, so to determine the coefficients, for the experiment times (the treatments) *n*, there must be $n\geq \frac{\left(m+1)(m+2\right)}{2}=15$. According to the orthogonal rotation central combination design, *n* can be defined as follows:

$$n=m_{c}+m_{\gamma }+m_{0}$$ (2)

Where *m*_*c*_ = 2^*m*^ is the experiment times of the orthogonal test with each factor at 2 levels (treatment 1–16 in Table 3), *m*_γ_ = 2*m* is the experiment times of the orthogonal test related to level γ (treatment 17–24 in Table 3), and *m*_0_ is the experiment times of the orthogonal test with each factor at 0 level, to maintain the rotary and orthogonality, γ and *m*_0_ can be defined as follows:

$$\gamma =\sqrt[{4}]{m_{c}}=2$$ (3)

$$m_{0}=\frac{(m+2){\left(m_{c}+2\;\gamma^{2}\right)}^{2}}{mm_{c}+2\;\gamma^{4}}-m_{c}-m_{\gamma }=12$$ (4)

Then the ranges of the four factors (N fertilizer (12.57–29.34 g plant^-1^); P fertilizer (1.14–2.65 g plant^-1^); K fertilizer (4.48–10.47 g plant^-1^); Water (7.20–16.80 L plant^-1^)) were determined based on experiences and knowledge of farmers, the corresponding actual value of level 0 (*a*_*v*_) is equal to the average value of the upper value and lower value of the ranges for each factor (Table 2), the changing interval (*Δ*_*j*_) between each actual value was calculated as follows:

$$\Delta_{j}=\frac{r_{a}}{2\;\gamma }$$ (5)

Where *r*_*a*_ is equal to the difference of the upper value and lower value of the ranges for each factor, and the corresponding actual value of level -1 and 1 can be calculated using the following formulas: a_v_−Δ_j_ and a_v_+Δ_j_. The four factors were N, P, and K fertilizers and water, given as *x*_*1*_, *x*_*2*_, *x*_*3*_, and *x*_*4*_, respectively, and there corresponding level given as *z*_1_, *z*_2_, *z*_3_, and *z*_4_,36 (n = 36) treatments in total (Table 3) were then used to find the fitting regression model that governs the effect of the four factors on the fruit yield and quality, and the coefficients in Eq (1) can be defined as follows:

$$a=\frac{1}{n}\sum_{i=1}^{n}y_{i}=\bar{y}$$ (6)

$${z_{ji}}^{\prime }=z_{ji}^{2}-\frac{1}{n}\sum_{i=1}^{n}z_{ji}^{2},j=1,2,\ldots ,m$$ (7)

$$b_{j}=\frac{\sum_{i=1}^{n}z_{ji}y_{i}}{\sum_{i=1}^{n}z_{ji}^{2}},j=1,2,\ldots ,m$$ (8)

$$b_{kj}=\frac{\sum_{i=1}^{n}{\left(z_{k}z_{j}\right)}_{i}y_{i}}{\sum_{i=1}^{n}{\left(z_{k}z_{j}\right)}_{i}^{2}},j>k,k=1,2,\ldots ,m-1$$ (9)

$$b_{jj}=\frac{\sum_{i=1}^{n}({z_{ji}}^{\prime })y_{i}}{\sum_{i=1}^{n}{\left({z_{ji}}^{\prime }\right)}^{2}},$$ (10)

Calcium nitrate (Ca (NO3)2·4H2O), sodium dihydrogen phosphate (NaH2PO4), and potassium sulfate (K2SO4), which were obtained from Shanghai Wintong Chemicals Co., Ltd. with a purity of more than 99%, were used as the sources of N, P, and K, respectively. Tap water was the source of water. All treatments were in a completely randomized block with three replications, and each treatment consisted of six plants. This give a total of 648 plants grown for the study in the experimental solar greenhouse. The 648 plants chosen for transplanting are all established, young runner plants that are about 2 months old with 3 to 4 leaves, and all plants got a mixture solution of the NPK fertilizers and water weekly according to the treatment arrangement 15 days after transplant, with application of additional macronutrients and micronutrients weekly with the same dosage for each treatment (Table 4). In addition, since the dosage of N fertilizer, P fertilizer, K fertilizer and water is very low for each plant, we calculated the dosage for one treatment (16 plants in total), then the mixed fertilizers and water was divided into 16 equal parts using graduated cylinder, and for dosage of the additional macronutrients and micronutrients (Table 4), we calculated the dosages for more treatments, mixed the fertilizers and water and divided them to small equal parts since it’s same for all treatments.

**Table 2:** Design level of four variables in the quadratic regression orthogonal experiment.

Variable X	Changing interval Δ_j_	Design level of variables (m_0_ = 12, γ = 2)				
		−γ	−1	0	+1	+γ
*x*_*1*_ (g plant^-1^)	13.41	12.57	16.77	20.96	25.15	29.34
*x*_*2*_ (g plant^-1^)	1.21	1.14	1.52	1.89	2.27	2.65
*x*_*3*_ (g plant^-1^)	4.78	4.48	5.98	7.48	8.97	10.47
*x*_*4*_ (L plant^-1^)	7.68	7.20	9.60	12.00	14.40	16.80

**Table 3:** Arrangement of variables in the quadratic regression orthogonal experiment and results of the experiment.

Treatments	*z*_*1*_	*z*_*2*_	*z*_*3*_	*z*_*4*_	*z*_*1*_*z*_*2*_	*z*_*1*_*z*_*3*_	*z*_*1*_*z*_*4*_	*z*_*2*_*z*_*3*_	*z*_*2*_*z*_*4*_	*z*_*3*_*z*_*4*_	*z*_*1*_^*’*^	*z*_*2*_^*’*^	*z*_*3*_^*’*^	*z*_*4*_^*’*^	Yield g plant^-1^	SSC %	TA %	SSC/TA
1	1	1	1	1	1	1	1	1	1	1	0.33	0.33	0.33	0.33	134.52	13.25	0.89	14.89
2	1	1	1	−1	1	1	−1	1	−1	−1	0.33	0.33	0.33	0.33	116.59	18.83	1.06	17.76
3	1	1	−1	1	1	−1	1	−1	1	−1	0.33	0.33	0.33	0.33	116.61	9.92	0.81	12.25
4	1	1	−1	−1	1	−1	−1	−1	−1	1	0.33	0.33	0.33	0.33	111.56	12.70	0.67	18.96
5	1	−1	1	1	−1	1	1	−1	−1	1	0.33	0.33	0.33	0.33	142.53	10.72	0.55	19.49
6	1	−1	1	−1	−1	1	−1	−1	1	−1	0.33	0.33	0.33	0.33	126.78	22.92	1.02	22.47
7	1	−1	−1	1	−1	−1	1	1	−1	−1	0.33	0.33	0.33	0.33	120.51	13.02	0.90	14.47
8	1	−1	−1	−1	−1	−1	−1	1	1	1	0.33	0.33	0.33	0.33	96.66	9.91	0.61	16.24
9	−1	1	1	1	−1	−1	−1	1	1	1	0.33	0.33	0.33	0.33	110.23	11.35	1.02	11.13
10	−1	1	1	−1	−1	−1	1	1	−1	−1	0.33	0.33	0.33	0.33	100.67	9.92	0.63	15.74
11	−1	1	−1	1	−1	1	−1	−1	1	−1	0.33	0.33	0.33	0.33	116.62	5.43	0.42	12.92
12	−1	1	−1	−1	−1	1	1	−1	−1	1	0.33	0.33	0.33	0.33	118.36	9.62	0.66	14.58
13	−1	−1	1	1	1	−1	−1	−1	−1	1	0.33	0.33	0.33	0.33	115.25	8.81	0.62	14.21
14	−1	−1	1	−1	1	−1	1	−1	1	−1	0.33	0.33	0.33	0.33	98.93	9.59	0.59	16.26
15	−1	−1	−1	1	1	1	−1	1	−1	−1	0.33	0.33	0.33	0.33	100.02	8.64	0.65	13.29
16	−1	−1	−1	−1	1	1	1	1	1	1	0.33	0.33	0.33	0.33	106.46	13.97	1.11	12.59
17	2	0	0	0	0	0	0	0	0	0	3.33	−0.67	−0.67	−0.67	126.51	11.59	0.85	13.64
18	−2	0	0	0	0	0	0	0	0	0	3.33	−0.67	−0.67	−0.67	96.70	7.79	0.62	12.56
19	0	2	0	0	0	0	0	0	0	0	−0.67	3.33	−0.67	−0.67	117.62	8.05	0.61	13.19
20	0	−2	0	0	0	0	0	0	0	0	−0.67	3.33	−0.67	−0.67	116.46	13.16	0.62	21.23
21	0	0	2	0	0	0	0	0	0	0	−0.67	−0.67	3.33	−0.67	127.43	13.83	0.67	20.64
22	0	0	−2	0	0	0	0	0	0	0	−0.67	−0.67	3.33	−0.67	107.63	9.24	0.47	19.67
23	0	0	0	2	0	0	0	0	0	0	−0.67	−0.67	−0.67	3.33	115.23	7.48	0.46	16.26
24	0	0	0	−2	0	0	0	0	0	0	−0.67	−0.67	−0.67	3.33	103.16	9.75	0.53	18.39
25	0	0	0	0	0	0	0	0	0	0	−0.67	−0.67	−0.67	−0.67	143.59	10.20	0.52	19.62
26	0	0	0	0	0	0	0	0	0	0	−0.67	−0.67	−0.67	−0.67	130.25	8.78	0.48	18.29
27	0	0	0	0	0	0	0	0	0	0	−0.67	−0.67	−0.67	−0.67	137.89	11.38	0.70	16.26
28	0	0	0	0	0	0	0	0	0	0	−0.67	−0.67	−0.67	−0.67	142.57	10.08	0.39	25.84
29	0	0	0	0	0	0	0	0	0	0	−0.67	−0.67	−0.67	−0.67	150.26	9.03	0.40	22.57
30	0	0	0	0	0	0	0	0	0	0	−0.67	−0.67	−0.67	−0.67	162.35	9.06	0.42	21.56
31	0	0	0	0	0	0	0	0	0	0	−0.67	−0.67	−0.67	−0.67	170.42	8.83	0.50	17.65
32	0	0	0	0	0	0	0	0	0	0	−0.67	−0.67	−0.67	−0.67	150.21	8.02	0.39	20.56
33	0	0	0	0	0	0	0	0	0	0	−0.67	−0.67	−0.67	−0.67	149.24	9.50	0.57	16.67
34	0	0	0	0	0	0	0	0	0	0	−0.67	−0.67	−0.67	−0.67	146.52	8.43	0.41	20.56
35	0	0	0	0	0	0	0	0	0	0	−0.67	−0.67	−0.67	−0.67	130.52	10.21	0.49	20.83
36	0	0	0	0	0	0	0	0	0	0	−0.67	−0.67	−0.67	−0.67	155.67	10.99	0.56	19.63

**Table 4:** Additional macronutrients and micronutrients applied to each treatment with the same dosage.

Fertilizers	Dosage (g plant^-1^)
MgSO_4_	4.43
EDTA-2NaFe	3.5461
H_3_BO_3_	0.3381
MnSO_4_·4H_2_O	0.2518
ZnSO_4_·7H_2_O	0.0260
CuSO_4_·5H_2_O	0.0095
(NH_4_)_6_Mo_7_O_24_·4H_2_O	0.0024

### Measurement of yield and fruit quality traits

An analytical balance (0.01 g accuracy) was used to measure the fruit weight after the fruits were harvested. Parameters of the fruit quality, namely, soluble solid content (SSC) and titratable acidity (TA), were measured after the fruits were transported to the laboratory. SSC (%) was determined by a digital hand-held pocket refractometer (PAL-1, Atago, Japan), whereas TA (%) was measured by neutralization to pH 7.0 with 0.1 N NaOH. Data are presented as percentages of malic acid.

### Software information

Data were processed by analysis of variance, using SPSS software version 21.0 and MATLAB software version R2017.

## Results

### Yield respond to N, P, and K fertilizers and water

After a significance test on regression coefficients and regression formulas, the equation that governs the effect of N (*x*_*1*_), P (*x*_*2*_), K (*x*_*3*_), and water (*x*_*4*_) on yield (*y*_*1*_) is determined according to Eqs (1)–(10):

$$\begin{array}{l} y_{1}=125.35+6.62x_{1}+0.85x_{2}+4.10x_{3}+4.35x_{4}-2.03x_{1}x_{2}+5.72x_{1}x_{3}+2.80x_{1}x_{4}\\ \;-3.81x_{2}x_{3}-1.17x_{2}x_{4}+2.43x_{3}x_{4}-8.85x_{1}{}^{2}-7.49xx_{2}{}^{2}-7.37x_{3}{}^{2}-9.45x_{4}{}^{2}\\ \;(R^{2}=0.87). \end{array}$$ (11)

Table 5 give the ANOVA results. The regression was significant at the 0.01 probability (F = 10.29 > F_0.01_ (14, 21) = 3.07), indicating that the regression model was a good fit for the experimental data. The F-value for the lack-of-fit test was 0.13. This value was less than the significant value at the 0.05 probability (F_0.05_ (10, 11) = 2.85), which was insignificant; the regression model was relatively suitable. Therefore, this regression model is valid to evaluate the effects of N, P, and K fertilizers on the ‘Hongyan’ strawberry yield.

**Table 5:** ANOVA table of effects of N (x1), P (x2), K (x3), and water (x4) on yield (y1).

Source of variance	*df*	*SS*	*MS*	*F-value*	*Significance*
*x*_*1*_	1.00	1051.26	1051.26	12.74	**
*x*_*2*_	1.00	17.24	17.24	0.21	ns
*x*_*3*_	1.00	402.62	402.62	4.88	*
*x*_*4*_	1.00	454.31	454.31	5.51	*
*x*_*1*_*x*_*2*_	1.00	65.69	65.69	0.80	ns
*x*_*1*_*x*_*3*_	1.00	522.81	522.81	6.34	*
*x*_*1*_*x*_*4*_	1.00	125.89	125.89	1.53	ns
*x*_*2*_*x*_*3*_	1.00	232.41	232.41	2.82	ns
*x*_*2*_*x*_*4*_	1.00	21.81	21.81	0.26	ns
*x*_*3*_*x*_*4*_	1.00	94.28	94.28	1.14	ns
*x*_*1*_^*2*^	1.00	2506.56	2506.56	30.38	**
*x*_*2*_^*2*^	1.00	1796.00	1796.00	21.77	**
*x*_*3*_^*2*^	1.00	1737.75	1737.75	21.06	**
*x*_*4*_^*2*^	1.00	2859.44	2859.44	34.66	**
Regression	14.00	11888.07	849.15	10.29	**
Residual	21.00	1732.65	82.51		
Lack of fit	10.00	183.29	18.33	0.13	ns
Error	11.00	1549.35	140.85		
Total	35.00	13620.72			

Abbreviations: df, degree of freedom; SS, Sum-of-squares; MS, mean squares.

F_0.05_ (1, 21) = 4.32, F_0.01_ (1, 21) = 8.02, F_0.05_ (14, 21) = 2.20, F_0.01_ (14, 21) = 3.07, F_0.05_ (14, 21) = 2.20, F_0.05_ (10, 11) = 2.85, F_0.01_ (10, 11) = 4.54.

ns, *, ** are not significant, significant at the 0.05 and 0.01 probability levels, respectively.

As shown in Table 5, N, K, and water had a significant effect on strawberry yield, but P had no significant effect. The relative magnitude of the effects of N, P, K, and water on yield was N>water>K>P in accordance with the absolute value of the standardized regression coefficient. No significant interaction occurred between N×P, N×water, P×K, P×water, and K×water. Thus, an ideal fit equation could be as follows:

$$\begin{array}{l} y_{1}=125.35+6.62x_{1}+4.10x_{3}+4.35x_{4}+5.72x_{1}x_{3}-8.85{x_{1}}^{2}-7.49{x_{2}}^{2}-7.37{x_{3}}^{2}-\\ \;9.45{x_{4}}^{2}. \end{array}$$ (12)

From the equation above, the partial regression equations were as follows:

$$y_{1}(x_{1})=125.35+6.62x_{1}-8.85{x_{1}}^{2},$$ (13)

$$y_{1}(x_{2})=125.35-7.49{x_{2}}^{2},$$ (14)

$$y_{1}(x_{3})=125.35+4.10x_{3}-7.37{x_{3}}^{2},$$ (15)

$$y_{1}(x_{4})=125.35+4.35x_{4}-9.45{x_{4}}^{2},$$ (16)

**Fig 3:**
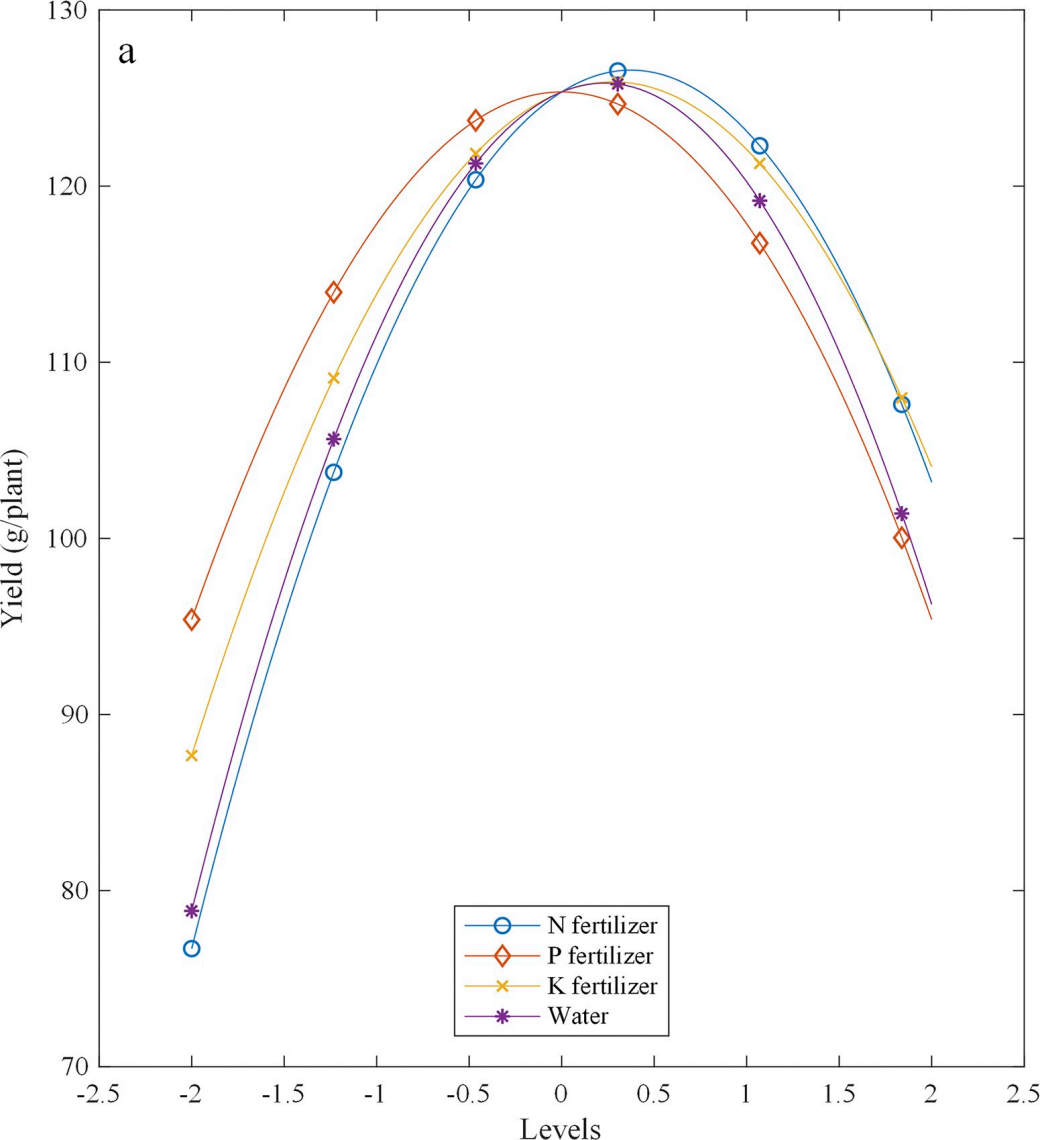
Effects of N, P, and K fertilizers and water on fruit yield.

The partial regression equation results showed that yield increased with an increase in N and P fertilizers at levels below 0.37 (22.51 g plant^-1^) and 0 (1.89 g plant^-1^), respectively, and decreased at levels above them (Fig 3). With increasing K fertilizer, the yield increased and then decreased, and it peaked at the 0.28 (7.90 g plant^-1^) level of K fertilizer. When increasing water, the yield increased and then gradually decreased, and the maximum value was at the 0.23 (12.55 L plant^-1^) level of water.

**Fig 4:**
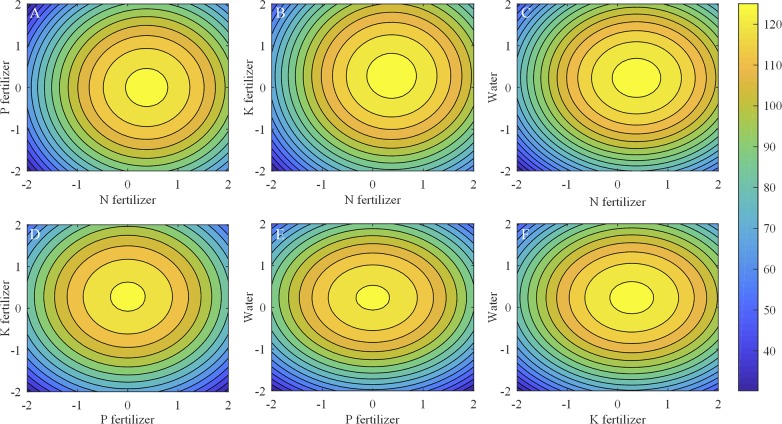
Effects of interaction among N, P, and K fertilizers and water on the yield. (A) N-P interaction effect on yield with K fertilizer and water at 0 level. (B) N-K interaction effect on yield with P fertilizer and water at 0 level. (C) N-water interaction effect on yield with P fertilizer and K fertilizer at 0 level. (D) P-K interaction effect on yield with N fertilizer and water at 0 level. (E) P-water interaction effect on yield with N fertilizer and K fertilizer at 0 level. (F) K-water interaction effect on yield with N fertilizer and P fertilizer at 0 level.

The interaction effect of every two factors on yield and SSC/TA ratio shows in Fig 4. Interaction analysis showed that yield increased and then decreased as the N fertilizer increased, but increased and then decreased as the P fertilizer increased. Furthermore, the maximum yield was 126.59 g plant^-1^ at 22.51 g plant^-1^ Ca (NO_3_)_2_·4H_2_O and 1.89 g plant^-1^ NaH_2_PO_4_ (Fig 4(A)). Yield increased and then decreased with increasing levels of combined N and K fertilizers, and the maximum yield was 127.16 g plant^-1^ at 22.51 g plant^-1^ Ca (NO_3_)_2_·4H_2_O and 7.90 g plant^-1^ K_2_SO_4_ (Fig 4(B)). The same trends were obtained for N×water interaction, that is, the yield increased then decreased, and reached the maximum yield of 127.09 g plant^-1^ at 22.51 g plant^-1^ Ca (NO_3_)_2_·4H_2_O and 12.55 L plant^-1^ water (Fig 4(C). Similarly, for the P×K (Fig 4(D), P×water (Fig 4(E), and K×water (Fig 4(F) interactions, the yield increased and then decreased, and the maximum yields were 125.92 g plant^-1^ at 1.89 g plant^-1^ NaH_2_PO_4_ and 7.90 g plant^-1^ K_2_SO_4_ (Fig 4(D), 125.85 g plant^-1^ at 1.89 g plant^-1^ NaH_2_PO_4_ and 12.55 L plant^-1^ water (Fig 4(E), and 126.42 g plant^-1^ at 7.90 g plant^-1^ K_2_SO_4_ and 12.55 L plant^-1^ water (Fig 4(F), respectively.

Horizontal axis and vertical axis are applied levels of the four factors, the corresponding actual values can be obtained according to Table 2, and the third axis (the colored contour line) are fruit yield (g plant^-1^).

We made frequency analysis to obtain the optimal fertilization combination for high yield (Table 6). Among 625 kinds of fertilization combinations, 27 combinations of the four factors had a yield of more than 110 g plant^-1^. The 99% confidence interval for N, P, and K fertilizers and water levels were 0.314–0.871, −0.357–0.357, 0.169–0.794, and 0.000–0.592, respectively. Therefore, when applying 22.28–24.61 g plant^-1^ Ca (NO_3_)_2_·4H_2_O, 1.75–2.03 g plant^-1^ NaH_2_PO_4_, 12.41–13.91 g plant^-1^ K_2_SO_4_, and 12.00–13.42 L plant^-1^ water, fruit yield will reach more than 110 g plant^-1^ with a probability of 99%.

**Table 6:** Frequency analysis of N, P, and K fertilizers and water for strawberry yield of more than 110 g plant^-1^.

Levels	N fertilizer		P fertilizer		K fertilizer		Water	
	ST	F	ST	F	ST	F	ST	F
−2	0	0.00	0	0.00	0	0.00	0	0.00
−1	0	0.00	7	0.26	1	0.04	2	0.07
0	12	0.44	13	0.48	13	0.48	15	0.56
1	14	0.52	7	0.26	12	0.44	10	0.37
2	1	0.04	0	0.00	1	0.04	0	0.00
Total	27	1	27	1	27	1	27	1
Average	0.593		0.000		0.481		0.296	
SE	0.108		0.139		0.121		0.115	
99% CI	0.314–0.871		−0.357–0.357		0.169–0.794		0.000–0.592	
OF (g plant^-1^)	22.28–24.61		1.75–2.03		12.41–13.91		12.00–13.42	

Abbreviations: ST, Sets Times; F, Frequency; SE, Standard Error; CI, confidence interval; OF, Optimal Fertilization.

### SSC/TA ratio responds to N, P, and K fertilizers and water

The regression equation that governs the effect on the SSC/TA ratio (*y*_*2*_) by N (*x*_*1*_), P (*x*_*2*_), K (*x*_*3*_), and water (*x*_*4*_) is determined according to Eqs (1)–(10):

$$\begin{array}{l} y_{2}=17.30+1.17x_{1}-1.12x_{2}+0.77x_{3}-1.09x_{4}-0.43x_{1}x_{2}+0.55x_{1}x_{3}-0.42x_{1}x_{4}\\ \;-0.94x_{2}x_{3}-0.61x_{2}x_{4}-0.19x_{3}x_{4}-1.97x_{1}{}^{2}-0.95x_{2}{}^{2}-0.21x_{3}{}^{2}-0.92x_{4}{}^{2}\\ \;(R^{2}=0.72). \end{array}$$ (17)

The ANOVA results show that the F-value for the regression model was 3.82, which was larger than F_0.01_ (14, 21) = 3.07 (Table 7). Thus, the regression model was a good fit for the experimental data. The F-value for the lack-of-fit test was 0.66. This value was less than the significant value at the 0.05 probability (F_0.05_ (10, 11) = 2.85), which was insignificant. Thus, the regression model was relatively suitable, is valid to evaluate the effects of N, P, and K fertilizers on the SSC/TA ratio of ‘Hongyan’ strawberry fruits.

**Table 7:** ANOVA table of effect of N, P, and K fertilizers and water on the SSC/TA ratio.

Source of variance	*df*	*SS*	*MS*	*F-value*	*Significance*
*x*_*1*_	1.00	32.60	32.60	5.46	*
*x*_*2*_	1.00	30.08	30.08	5.04	*
*x*_*3*_	1.00	14.40	14.40	2.41	ns
*x*_*4*_	1.00	28.62	28.62	4.80	*
*x*_*1*_*x*_*2*_	1.00	2.92	2.92	0.49	ns
*x*_*1*_*x*_*3*_	1.00	4.76	4.76	0.80	ns
*x*_*1*_*x*_*4*_	1.00	2.81	2.81	0.47	ns
*x*_*2*_*x*_*3*_	1.00	14.12	14.12	2.37	ns
*x*_*2*_*x*_*4*_	1.00	5.94	5.94	1.00	ns
*x*_*3*_*x*_*4*_	1.00	0.59	0.59	0.10	ns
*x*_*1*_^*2*^	1.00	124.81	124.81	20.92	**
*x*_*2*_^*2*^	1.00	28.72	28.72	4.81	**
*x*_*3*_^*2*^	1.00	1.43	1.43	0.24	ns
*x*_*4*_^*2*^	1.00	27.01	27.01	4.53	*
Regression	14.00	318.81	22.77	3.82	**
Residual	21.00	125.31	5.97		
Lack of fit	10.00	47.05	4.70	0.66	ns
Error	11.00	78.26	7.11		
Total	35.00	456.55			

F_0.05_ (1, 21) = 4.32, F_0.01_ (1, 21) = 8.02, F_0.05_ (14, 21) = 2.20, F_0.01_(14, 21) = 3.07, F_0.05_ (14, 21) = 2.20, F_0.05_ (10, 11) = 2.85, F_0.01_ (10, 11) = 4.54.

ns, *, ** are not significant, significant at the 0.05 and 0.01 probability levels, respectively.

The N and P fertilizers and water had a significant effect on the SSC/TA ratio of the strawberry fruit, but the K fertilizer had no such effect. The relative magnitude of the effects of N, P, and K fertilizers and water on the SSC/TA ratio was N>P>water>K, which was in accordance with the absolute value of the standardized regression coefficient. No significant interaction occurred among N, P, K, and water in terms of the SSC/TA ratio (Table 7). Therefore, an ideal fit equation could be as follows:

$$y_{2}=17.30+1.17x_{1}-1.12x_{2}-1.09x_{4}-1.97{x_{1}}^{2}–0.95{x_{2}}^{2}-0.92{x_{4}}^{2}.$$ (18)

From the equation above, the partial regression equations were as follows:

$$y_{2}(x_{1})=17.30+1.17x_{1}-1.97{x_{1}}^{2},$$ (19)

$$y_{2}(x_{2})=17.30-1.12x_{2}–0.95{x_{2}}^{2},$$ (20)

$$y_{2}(x_{4})=17.30-1.09x_{4}-0.92{x_{4}}^{2}.$$ (21)

**Fig 5:**
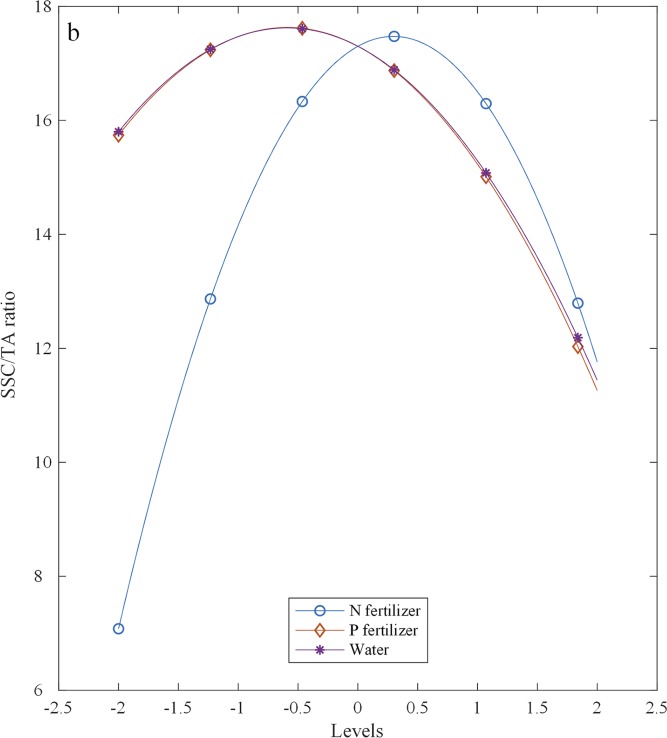
Effects of N, P, and K fertilizers and water on the SSC/TA ratio.

The partial regression equation results showed that the SSC/TA ratio increased with an increase in P and water at levels below −0.59 (1.67 g plant^-1^) and −0.59 (10.58 g plant^-1^), respectively, and decreased at levels above them (Fig 5). With increasing N, the SSC/TA ratio gradually increased and then gradually decreased, with a maximum value at the 0.30 (22.22 g plant^-1^) level of N.

**Fig 6:**
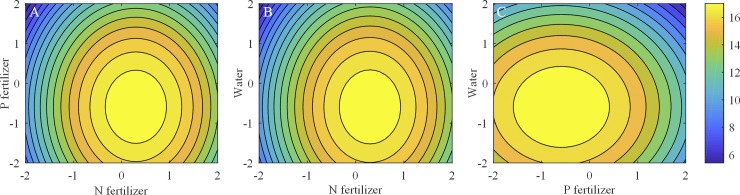
Effects of the interaction among N, P, and K fertilizers and water on the SSC/TA ratio. (A) N-P interaction effect on SSC/TA ratio with water at 0 level. (B) N-water interaction effect on SSC/TA ratio with P fertilizer at 0 level. (C) P-water interaction effect on SSC/TA ratio with P fertilizer at 0 level. Horizontal axis and vertical axis are applied levels of the four factors, the corresponding actual values can be obtained according to Table 2, and the third axis (the colored contour line) stands for SSC/TA ratio.

The SSC/TA ratio increased and then decreased with increasing levels of N, whereas it increased and then decreased with increasing P; meanwhile, the maximum SSC/TA ratio was 17.80 at 22.22 g plant^-1^ Ca (NO_3_)_2_·4H_2_O and 1.67 g plant^-1^ NaH_2_PO_4_ (Fig 6(A). The same trend in Fig 6(A) was obtained in Fig 6(B) for the N×water interaction, and the maximum SSC/TA ratio was 17.80 at 22.22 g plant^-1^ Ca (NO_3_)_2_·4H_2_O and 10.58 L plant^-1^ water (Fig 6(B). For the P×water interaction, the SSC/TA ratio increased and then decreased with increasing P fertilizer and water, and the maximum SSC/TA ratio was 17.64 at 1.67 g plant^-1^ NaH_2_PO_4_ and 10.58 L plant^-1^ water (Fig 6(C).

We performed frequency analysis to obtain the optimal fertilization combination for preferable SSC/TA ratio (Table 8). Among 625 fertilization combinations, 47 combinations of the three factors were available with SSC/TA ratio of strawberry fruits varying between 8.5 and 14. The 99% confidence interval for N, P, and water levels were 0.101–0.962, −0.663–0.407, and −0.650–0.437, respectively. Thus, when applying 21.38–24.99 g plant^-1^ Ca (NO_3_)_2_·4H_2_O, 1.64–2.04 g plant^-1^ NaH_2_PO_4_, and 10.44–13.05 L plant^-1^ water, the fruit SSC/TA ratio will reach 8.5–14 with a probability of 99%.

**Table 8:** Frequency analysis of N, P, and water for strawberry fruit’s SSC/TA ratio of 8.5–14.

Levels	N fertilizer		P fertilizer		Water	
	ST	F	ST	F	ST	F
−2	0	0.00	12	0.26	12	0.26
−1	12	0.26	8	0.17	8	0.17
0	11	0.23	8	0.17	8	0.17
1	11	0.23	12	0.26	11	0.23
2	13	0.28	7	0.15	8	0.17
Total	47	1	47	1	47	1
Average	0.532		−0.128		−0.106	
SE	0.167		0.208		0.211	
99% CI	0.101–0.962		−0.663–0.407		−0.650–0.437	
OF (g plant^-1^)	21.38–24.99		1.64–2.04		10.44–13.05	

Abbreviations: ST, Sets Times; F, Frequency; SE, Standard Error; CI, confidence interval; OF, Optimal Fertilization.

### Optimal fertilization (OF) combination for both high yield and best quality

In accordance with the intersection calculations of the optimal fertilization combination for high yield and best quality, the best fertilization combination for high yield (more than 110 g plant^-1^) and best fruit SSC/TA ratio (8.5–14), were 22.28–24.61 g plant^-1^ N, 1.75–2.03 g plant^-1^ P, 12.41–13.91 g plant^-1^ K, and 12.00–13.05 L plant^-1^ water, where N, P, K stands for Ca (NO_3_)_2_·4H_2_O, NaH_2_PO_4_, K_2_SO_4_ respectively. In addition, due to the limitation of the experiment design, some of the nutrient combination applied to the treatments that reach ‘stress’ levels will cause quality change of the berries in size, shape, and taste, this may had affected the analysis of the result.

## Discussion

Currently, market intermediaries pay considerable attention to fruit quality to enhance profits by meeting the consumer preference of sweetness [11,45]. The SSC/TA ratio has been widely used as a reliable predictor to evaluate the strawberry combined taste of sweetness and sourness. Strawberries are sweeter if their SSC/TA ratio is high than if their SSC/TA ratio is low [12,15,28,46,47]. The minimum SSC/TA ratio for acceptable combined taste is 8.75 [48], and people prefer the taste of cultivars ‘Clery’ and ‘Daroyal’, which have high SSC/TA ratios of 9.66 and 9.26, respectively [49]. The cultivar ‘NCS 10–156’ has an SSC/TA ratio of 11.6, and it is believed to be more suitable for sale than other cultivars [50]; all these SSC/TA ratios are consistent with the typical range (8.5–14) for strawberries with optimal fruit quality [51,52]. In general, the SSC/TA ratio is an important parameter to evaluate fruit quality for strawberry production [53,54]. Therefore, the SSC/TA ratio was the focus of this study.

Previous studies have shown that N, P, K, and water have significant effects on the yield and fruit quality of strawberry [27,55–58]. We used a quadratic regression orthogonal rotation combination experimental design to investigate the optimal fertilization and water combination for high strawberry yield and best fruit quality (optimal SSC/TA ratio). In the present work, N, P, and water had significant effects on yield and SSC/TA ratio, whereas K had a significant effect on yield only. Except for the interaction between N and K having a significant effect on yield, the other interactions among the four factors showed no differences concerning yield and SSC/TA ratio. The effects of the four factors on yield and SSC/TA ratio were ranked as N>water>K>P and N>P>water>K, respectively. N was the most important factor of the four factors, and showed significant effect on yield and SSC/TA ratio. Therefore, N was the key factor in determining fruit yield and quality. By contrast, if application levels were above 0, P and water had a significant negative effect on the SSC/TA ratio; this result is comparable with findings of others [27,59]. Excessive P suppresses SSC production and promotes TA formation, while excessive water reduces the fruits’ sweetness perception.

The combined application of fertilizer and water should optimize based on the interaction analysis in the present study. The yield and SSC/TA ratio increased and then decreased as two of the factors increased, while the two other factors were fixed at 0 level. These trends indicated maximum or optimal values of yield and SSC/TA ratio.

The optimal fertilizer and water combination for high yield (>110 g plant^-1^) and best fruit quality (SSC/TA ratio of 8.5–14) was achieved by using a quadratic regression orthogonal rotation combination experimental design and variance analysis. the optimal fertilizer and water combination was found to be 22.28–24.61 g plant^-1^ Ca (NO_3_)_2_·4H_2_O, 1.75–2.03 g plant^-1^ NaH_2_PO_4_, 12.41–13.91 g plant^-1^ K_2_SO_4_, and 12.00–13.05 L plant^-1^ water.

Although we achieved a good result with the statistical method, we only choose yield and SSC/TA ratio as the main index without consider that fruit weight or size may also affect the fruit price in market, and multiple years’ experiments should be conducted to validate the result in this paper, we will surely consider these factors in our further researches.

## Conclusion

N was the most important of four factors that had a significant effect on both yield and SSC/TA ratio, followed by water and P. Nitrogen, P, and water significantly influence on yield and SSC/TA, whereas K had a significant effect only on yield. The N×K interaction had a significant effect on yield. However, the other interactions among the four factors showed no significant effects on yield and SSC/TA. The effects of the four factors on the yield and SSC/TA ratio were ranked as N>water>K>P and N>P>water>K, respectively. The yield and SSC/TA ratio increased and thereafter decreased when NPK fertilizers and water increased. The optimal fertilizer and water combination for high yield (>110 g plant^-1^) and best fruit quality (SSC/TA ratio of 8.5–14) was 22.28–24.61 g plant^-1^ Ca (NO_3_)_2_·4H_2_O, 1.75–2.03 g plant^-1^ NaH_2_PO_4_, 12.41–13.91 g plant^-1^ K_2_SO_4_, and 12.00–13.05 L plant^-1^ water. We consider the present results useful for further research on fertilization and water application to further improvement of crops.

## Supporting information

S1 Dataset Regression analysis of the effect of the four factors on yield and SSC/TA ratio. (XLSX)
